# Monocytes from people with Familial Hypercholesterolaemia are inflammatory, despite statin-treatment

**DOI:** 10.1016/j.athplu.2025.09.002

**Published:** 2025-09-10

**Authors:** Helen Williams, Habib Francis, Jasmin Huang, Rekha Marimuthu, Rana Baraz, Heather Medbury, Stephen Li

**Affiliations:** a Vascular Biology Research Centre, Department of Surgery, WSLHD Research and Education Network Building, Westmead Hospital, Hawkesbury Road, Westmead, NSW, Australia; b Sydney Medical School, The University of Sydney, Westmead, NSW, 2145, Australia; c Chemical Pathology, NSW Health Pathology, Westmead Hospital and Institute of Clinical Pathology and Medical Research, Hawkesbury Road, Westmead, NSW, 2145, Australia; d Blacktown/Mt Druitt Clinical School, Blacktown Hospital, Western Sydney University, Blacktown Road, Blacktown, NSW, 2148, Australia

**Keywords:** Cholesterol, Monocytes, Inflammation, Macrophages, Atherosclerosis, Statins

## Abstract

**Background and aims:**

Familial Hypercholesterolaemia (FH) is characterised by high cholesterol and premature cardiovascular disease. While hypercholesterolaemia and inflammation are both key drivers in the formation of atherosclerotic plaques, inflammation remains understudied in FH. Inflammatory (M1) macrophages contribute to plaque destabilisation and macrophage precursors, monocytes, can be skewed towards an inflammatory state. Aims: Determine; whether monocytes of FH individuals are inflammatory, if they readily form inflammatory macrophages, and whether this remains so in statin-treated individuals.

**Methods:**

Blood samples were collected from people with FH (statin-treated and untreated) and healthy controls. Lipid profile was obtained and monocyte inflammatory marker expression was determined by whole blood flow cytometry. Monocytes were cultured with autologous serum and resultant macrophage profile determined by flow cytometry.

**Results:**

Total cholesterol and low-density lipoprotein cholesterol (LDL-C) were higher in the Untreated-FH group compared to the Treated-FH group and controls. In both Treated-FH and Untreated-FH groups, monocytes were inflammatory with high CD86 (M1). The ratio of inflammatory/anti-inflammatory markers (CD86/CD163) significantly correlated with LDL-C and ApoB/ApoA1 ratio across the cohort, indicating the high LDL-C of FH may promote an inflammatory monocyte profile. Monocyte-derived-macrophages from (Treated) FH individuals also had a more inflammatory profile (CD86 and CD86/CD163).

**Conclusions:**

Overall, monocytes show inflammatory skewing in FH individuals, even those with moderately-reduced cholesterol levels. These monocytes readily become inflammatory macrophages. This, along with subsequent inflammatory macrophage formation, could contribute to plaque destabilisation and downstream clinical events. This supports inflammatory monocyte targeting as a potential approach to reduce residual risk in FH individuals.

## Highlights

• Monocytes are inflammatory in FH individuals, even in statin-treated group.
• Monocyte-derived-macrophages were more inflammatory in FH than controls.
• Monocyte M1 skewing associated with elevated LDL-C levels.
• Inflammatory monocytes/macrophages indicates residual inflammation in FH.

## Graphical abstract

**Figure:**
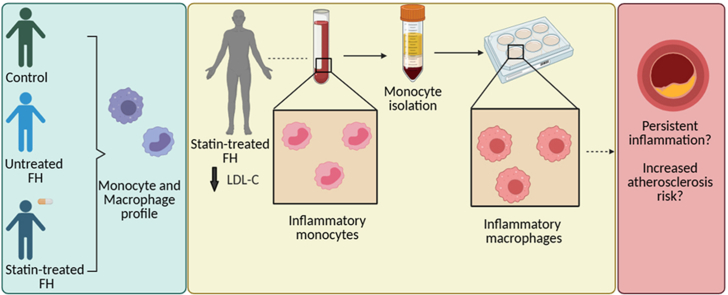

## 1 Introduction

Familial hypercholesterolaemia (FH) is a disease resulting from inherited mutations in cholesterol clearance pathway genes [1]. This results in greatly elevated low-density lipoprotein cholesterol (LDL-C) levels and premature cardiovascular disease (CVD) [2]. Even in the era of statins, FH individuals aged 50–70 have double the incidence of acute myocardial infarction (AMI) as age-matched individuals without FH. In people aged 25–39 the incidence rate is over seven times higher in FH individuals than those without FH [3]. As LDL-C retention and oxidation initiate atherosclerotic plaque formation [4], the elevated LDL-C in FH individuals drives atherosclerosis [5]. Accordingly, substantially lowering LDL-C can reduce the overall cardiovascular event risk [6]. Unfortunately, despite lipid-lowering, major cardiovascular events continue to occur, likely because cholesterol is not the only factor driving CVD. Inflammation has been recognised as a key player for two decades [7]. Indeed, the contribution of inflammation (measured by high sensitivity C-reactive protein [hsCRP]) to future cardiovascular events is similar to that of hypercholesterolaemia, and in statin-treated groups, inflammation is a stronger predictor than cholesterol levels [8]. The link between inflammation and clinical outcome is not limited to hsCRP, with immune cells being key players in inflammatory response [9]. For example, an elevation in ‘inflammatory’ monocytes is associated with cardiovascular death, AMI, and stroke [10]. This link makes sense as, within plaques, monocyte-derived-macrophages perform a myriad of functions that can influence plaque stability. While the M1/M2 classification of macrophages, M1 being inflammatory and M2 being healing, is simplistic, it nevertheless remains relevant to plaque stability [11]. In human carotid atherosclerosis plaques, presence of CD86^+^ (M1) macrophages, and the ratio of CD86/CD163 (M1/M2), were negatively correlated with cap thickness, indicating these inflammatory macrophages were associated with instability [12]. Recently, microarray and RNA sequencing data showed that both M1 and M2 macrophages are present in unstable plaques, with M1 macrophages strongly associated with, and even a key cause of, instability [13].

The inflammatory state of macrophages could be influenced by the state of their precursors, monocytes. Monocytes from people with CVD are inclined towards an M1 macrophage state, with high CD86/CD163 (M1/M2) ratio [14]. Further, in generally healthy people, CD86/CD163 ratio on monocytes correlated negatively with high density lipoprotein cholesterol (HDL-C), indicating that lipid levels may influence monocyte profile [14]. Such monocyte alterations do appear to influence macrophage phenotype, with inflammatory monocyte skewing in obesity accompanied by reduced formation of M2-like macrophages in vitro [15]. Thus, FH with its high LDL-C levels could indeed promote monocyte inflammatory (M1) marker expression, enhance M1 macrophage formation, and thereby contribute to plaque instability. There are few studies investigating this. One such study reported a greater percentage of ‘M1 monocytes’ (CD68+CCR2+) in FH [16]. However, this FH population was predominantly (95 %) statin-treated and a comparator of untreated FH was not included. Considering inflammation in FH may persist despite lipid-lowering treatment [17], this comparison is important. Further, the study assessed the percentage of ‘M1 monocytes’ rather than skewing (i.e. the degree of marker expression). Examining M1 skewing on monocytes, using a wider range of phenotypic markers (such as CD86 which is associated with unstable plaques), would provide further information on the nature of inflammatory monocyte/macrophage changes in FH. Moreover, understanding if monocyte M1 marker expression is associated with subsequent adoption of an M1 macrophage phenotype would provide new insight into whether an inflammatory monocyte profile could be an important risk factor for FH individuals. We hypothesise; that monocytes are M1 skewed in FH, that this persists despite statin treatment, and that this will result in greater formation of M1 macrophages from FH monocytes.

## 2 Materials and methods

### 2.1 Participants and sample size

Written informed consent was obtained from each participant included in the study. The study protocol conforms to the 1975 Declaration of Helsinki and was approved by the Western Sydney Local Health District (WSLHD) Human Research Ethics Committee (AU RED HREC/15/WMEAD/289). Adults (>18 years) of any sex were recruited between 2016 and 2021 in two groups; probable FH individuals and healthy controls. CONSORT diagram of cohort recruitment is shown in Supplementary Fig. 1. Controls were invited to participate if they were generally healthy and not taking lipid lowering medications. Probable FH participants were selected from people with dyslipidaemia attending the Westmead Hospital Lipid Clinic. Inclusion criteria for FH group was a Dutch Lipid Clinic Network Score of at least 6 (probable FH). Medical history was obtained from all participants and included: self-reported demographic information (age, sex, height, weight), medications (lipid lowering, diabetes or blood pressure), physical indicators of FH (e.g. tendon xanthomas), CVD risk factors (high blood pressure, diabetes, smoking, inflammatory conditions), and CVD history (e.g. AMI, angina, or stroke). Exclusion criteria for Control group was; diagnosed CVD, hypertension, or diabetes mellitus (Type 1 or 2). The FH group was subdivided into statin-treated (Treated-FH) and untreated (Untreated-FH) groups based on current medications.

Sample size was calculated using G∗Power (3.1) [18,19]. Considering that a similar study [16] found large differences in proportion of ‘M1 monocytes’ (effect size f estimated as 0.7), we set a large effect size (f = 0.7) with 80 % power at a significance level (alpha) of 0.05. This gave a required sample size of n = 24 (i.e. 8 per group). For correlations, using all participants, moderate strength associations could be detected with n = 26 (Pearson correlation coefficient 0.5). We therefore required a minimum of n = 8 per group and a total of n = 26 in the study to detect large differences between groups and moderate correlations between factors.

### 2.2 Biochemical and lipid measurements

Peripheral blood samples were collected from all participants. White blood cell count and differential were performed on automated cell counters. Total cholesterol (TC), HDL-C, LDL-C, triglycerides, apolipoprotein (Apo) A1, ApoB, and CRP were measured using enzymatic colorimetric assay (lipid profile) or immunoturbidimetric assay (Apo and CRP) on Roche Cobas Pro by the ICPMR-Westmead, NSW Health Pathology. Participants with CRP value > 10 mg/L, indicative of a current inflammatory condition, were excluded. Control participants with hypercholesterolemia (LDL-C > 3.5 mmol/L) were excluded.

### 2.3 Monocyte flow cytometry

Whole blood flow cytometry was used to examine monocyte subset proportions and M1/M2 marker expression. Antibodies were titrated to determine appropriate concentrations, and fluorophore panel was selected to minimise spectral overlap. Fresh K2EDTA (BD, NSW, Australia) anti-coagulated blood (50 μL) was stained with anti-CD14-V450 (BD, NSW, Australia, Cat#560349), anti-CD16-APC (R&D Systems, Minneapolis, MN, Cat# FAB2546A) and anti-HLA-DR-Per-CP (Biolegend, San Diego, CA, Cat#307628) to identify monocyte subsets. PE-conjugated M1 and M2 markers or their corresponding isotype controls, were each assessed in separate tubes. M1 markers were: CD64 (BD Cat#561926), CD86 (BD Cat#560957), and CD120b (R&D Systems, Cat#FAB226P). M2 markers were: CD11b (BD Cat# 561001), CD93 (Biolegend, San Diego, CA Cat# 336107), and CD163 (BD Cat# 556018). All markers showed measurable expression in monocytes against isotype controls (Supplementary Fig. 2). Staining was for 30 min, protected from light. Cells were subsequently fixed and red blood cells lysed by the addition of 250 μL Optilyse C (Beckman coulter, NSW, Australia). Data was acquired on a BD FACS™ Canto II flow cytometer (BD, NSW, Australia) using FACSdiva software (v6.0, BD, NSW, Australia). The gating strategy was previously published (Supplementary Fig. 3) [20]. Approximately 2500 events in the final monocyte gate (Supplementary Fig. 3E) were recorded. Data analysis was performed using FlowJo™ v10.9.0 (BD Life Sciences) with researcher blinded to the sample study group. The relative level of marker expression was determined by the ratio of the median fluorescence intensity (MFI) of the marker of interest over the MFI of the isotype control.

### 2.4 Monocyte-derived-macrophage flow cytometry

EDTA-anticoagulated blood was diluted with equal volumes of PBS and overlayed on lymphoprep (Stemcell Technologies, VIC, Australia). Peripheral blood mononuclear cells (PBMCs) were obtained from the interface following centrifugation (800×*g*, 20 min, RT). Monocytes were isolated from PBMCs using monocyte isolation kit without CD16 depletion (Cat#19058, Stemcell Technologies, VIC, Australia), as per manufacturer instructions. Blood collected in serum separator tubes was allowed to clot for at least 30 min. Tubes were centrifuged (1300×*g*, 10 min, RT) and serum collected. Monocytes (0.4 × 10^6^ cells/mL) were then cultured in RPMI (Lonza cat#BE12-702F) with 20 % autologous serum for a total of 7 days. RPMI + serum was changed at 24 h to remove nonadherent cells, then subsequently replaced each 2–3 days. Macrophages were detached by incubation with accutase (Stemcell Technologies, VIC, Australia) for 20 min at 37 °C 5 % CO_2_ following manufacturer's directions, and then retrieved by pipetting up and down. Cells were incubated with antibodies against CD86 and CD163 and marker expression was assessed by flow cytometry. Some samples were excluded from this analysis due to technical issues isolating or staining the cells. Corrected numbers for this section were Control n = 8, Untreated-FH n = 8, and Treated-FH n = 10.

### 2.5 Serum cytokine analysis

Serum was collected as above and stored at −80 °C until analysed. Concentrations of cytokines (IFN-γ, IL-1β, IL-1RA, IL-4, IL-6, IL-8, IL-10, IL-12, IL-17, MCP-1 and TNF-α) were determined by Luminex HCYTOMAG60-K (Merck Life Science, VIC, Australia) following manufacturer's instructions. Briefly, quality controls, standards or serum along with cytokine beads were added to a 96-well plate and incubated overnight. The plate was attached to a magnet and washed. Detection antibodies were added, and the plate was incubated for 1 h, followed by addition of Streptavidin-Phycoerythrin and a further 30-min incubation. The plate was then washed on a magnet and sheath fluid added to all wells before being read on a Luminex™ 200™ system using xPONENT® software (Thermo Fisher Scientific, Australia). The average mean fluorescence intensity of each cytokine was recorded for each sample and the concentration was automatically calculated based on the standard curve obtained.

### 2.6 Statistical analysis

SPSS software (Version 29.0.1.0 (171), IBM, Armonk, NY) and GraphPad Prism 10.2.3 (GraphPad Software, Boston, MA) were used for statistical analysis and data visualisation. For continuous data, normality was queried by use of Shapiro-Wilk and Anderson-Darling tests. If these disagreed, boxplots were scrutinised to assess distribution. Normally distributed data were shown as mean ± standard deviation (SD) and non-normal data as median (Quartile 1, Quartile 3). Comparisons between groups were performed using ANOVA followed by post hoc Tukey's or Dunnet's multiple comparisons test for normally distributed datasets and by Kruskall Wallis with post hoc Dunn's multiple comparison test for non-normally distributed datasets. All tests were two-tailed. For comparisons of categorical data (e.g. sex, tendon xanthomas), Fisher's exact test was used. For all comparisons between groups, *p<0.05* was considered statistically significant. Associations between data were assessed with Pearson's correlation, or Spearman's Rho where data were non-normally distributed. Bonferroni correction was applied to *p-values* to adjust for multiple comparisons.

## 3 Results

### 3.1 Lipid profile is improved in Treated-FH group

From an initial 47 participants, this cohort included 11 controls, 8 Untreated FH participants and 11 Treated FH participants (Supplementary Fig. 1). Full details of lipid-lowering medication taken by Treated FH group are shown in Supplementary Table 1. Five subjects were on rosuvastatin, three on atorvastatin and three on simvastatin. Of these, four patients were also on ezetimibe (10 mg/day). Note that treated group were all taking current statin >2 months. The three study groups (Treated-FH, Untreated-FH and Control) were well-matched with a similar mean age and body mass index. All groups showed an equivalent predominance of females (Table 1)*.* The groups were matched in their CVD history, with no participants in any group reporting CVD history, diabetes or hypertension and only one current smoker across the entire cohort. Over half the FH participants reported tendon xanthomas while, naturally, none of the controls did (Table 1). Compared to controls, the Untreated-FH participants had significantly higher TC, LDL-C, cholesterol/HDL ratio, and ApoB. Consequently, the ApoA1/ApoB ratio was significantly lower while the ApoB/ApoA1 ratio was higher (Table 1). Differences between Treated-FH group and controls were not detected. Compared to the Untreated-FH group, the Treated-FH group had significantly lower TC (−37 %, *p<0.004*), LDL-C (−44 %, *p<0.013*), ApoB (−36 %, *p<0.001*), and triglycerides (−37 %, *p<0.026*).

**Table 1:** Participant demographics.

	Control (n = 11)	Untreated FH (n = 8)	Treated FH (n = 11)	Global *p =*
Sex Female (number, %)	8 (73 %)	6 (75 %)	8 (73 %)	>0.999
Age (years)	36 [23–58]	44 [30–63]	37 [18–76]	0.623
Body Mass Index (kg/m^2^)	24.4 ± 2.3	26.1 ± 4.8	26.4 ± 4.0	0.412
Xanthomas n (%)	0 (0 %)	**5 (63 %)∗∗**	**8 (73 %)∗∗**	**<0.001**
CVD n (%)	0 (0 %)	0 (0 %)	0 (0 %)	>0.999
Smokers n (%)	0 (0 %)	1 (12.5 %)	0 (0 %)	>0.999
Diabetes n (%)	0 (0 %)	0 (0 %)	0 (0 %)	>0.999
Hypertension n (%)	0 (0 %)	0 (0 %)	0 (0 %)	>0.999
TC (mM)	4.8 (3.7, 4.95)	**7.9 (7.65, 8.75)∗∗∗**	**5.3 (4.65, 5.65) ††**	**<0.001**
LDL (mM)	2.6 (1.9, 2.75)	**5.5 (4.85, 6.2)∗∗∗**	**3.1 (2.5, 3.25) †**	**<0.001**
HDL (mM)	1.60 ± 0.47	1.86 ± 0.25	1.63 ± 0.45	0.376
Chol/HDL	2.6 (2.45, 2.95)	**4.65 (3.85, 4.91)∗∗**	**3.0 (2.84, 3.7) †**	**0.005**
Apo A1 (g/L)	1.59 ± 0.42	1.83 ± 0.23	1.66 ± 0.33	0.334
Apo B (g/L)	0.77 ± 0.25	**1.58** ± **0.37∗∗∗**	**1.01** ± **0.23†††**	**<0.001**
Apo A/B	2.06 (1.82,2.34)	**1.20 (1.01, 1.41)∗∗**	1.72 (1.48, 1.92)	**0.006**
Apo B/A1	0.48 (0.43, 0.55)	**0.83 (0.71, 0.98)∗∗**	0.58 (0.52, 0.68)	**0.006**
Triglyceride (mM)	1.0 (0.64, 1.33)	1.63 (1.29, 1.78)	**0.93 (0.78, 1.14) †**	0.063

Except for age, which is presented as median [range], continuous data are presented as mean ± SD for normally distributed data or median (Q1, Q3) for non-normally distributed data. Nominal data is presented as number (%). ANOVA followed by post hoc Tukey's test was used for normally distributed datasets and Kruskall Wallis with Dunn's post hoc was used for non-normally distributed datasets. Bolded values indicate significant difference against another group ∗ indicates *p<0.05*, ∗∗*p<0.01*, ∗∗∗*p<0.001* vs. control. † indicates *p<0.05*, ††*p<0.01*, †††*p<0.001* vs. FH-Untreated. Abbreviations: TC, total cholesterol; LDL, low-density lipoprotein; HDL-C, high-density lipoprotein cholesterol; Apo, apolipoprotein; SD, standard deviation.

### 3.2 Monocyte M1 marker (CD86) is higher in FH

**Fig. 1:**
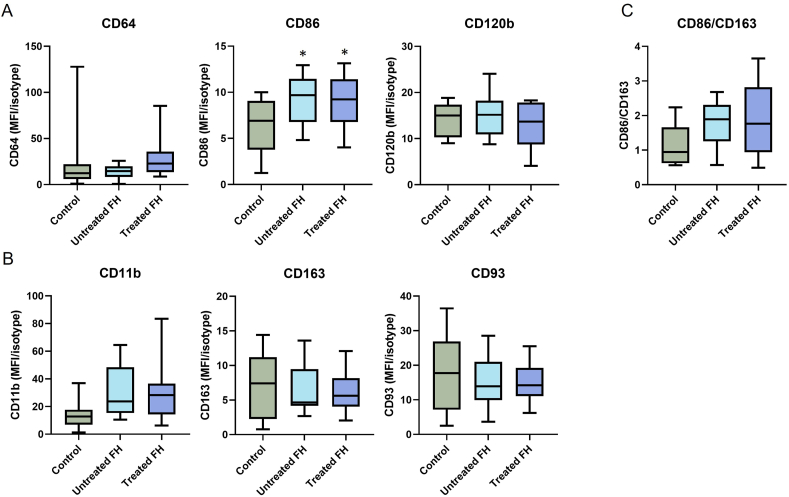
Monocyte M1 and M2 marker expression. Marker expression on all monocytes was determined by whole blood flow cytometry (Control n = 11, Untreated FH n = 8, Treated FH n = 11). Markers are represented as median fluorescence intensity (MFI) of marker over MFI of isotype. Data are shown as box-and-whisker plots. **(A)** M1 markers (CD64, CD86, CD120b) **(B)** M2 markers (CD11b, CD93, CD163) (**C)** CD86/CD163 ratio. ANOVA followed by post hoc Dunnet's or Tukey's multiple comparison test was used for normally distributed datasets and Kruskall Wallis followed by post hoc Dunn's multiple comparison test was used for non-normally distributed datasets. ∗*p<0.05* vs. control.

White blood cell profile did not show differences between groups (Table 2). Proportions of monocyte subsets (classical, intermediate and nonclassical) were similar across the groups, with all showing predominance of the classical monocytes (∼85 %) with fewer intermediates (∼6 %) and nonclassicals (∼9 %). The inflammatory M1 marker, CD86, differed between groups, being ∼50 % higher in both FH groups compared to controls (*p = 0.044* for both). Other M1 markers (CD64 and CD120b) did not differ between groups (Fig. 1, *p = 0.201*, *p = 0.594*, respectively). The M2 markers CD93 and CD163 did not differ between groups (*p = 0.689*, *p = 0.803*, respectively). CD11b appeared higher in FH groups compared to controls but this was not a significant difference (*p = 0.050*, *p = 0.078,* respectively). The ratio of the M1/M2 markers (CD86/CD163) trended higher in the FH groups but did not significantly differ between groups (*p = 0.114*). In serum, none of the eight M1-related or three M2-related cytokines were found to differ between groups (Supplementary Table 2)

**Table 2:** White blood cell counts.

	Control (n = 11)	Untreated FH (n = 8)	Treated FH (n = 11)	Global *p=*
WBC (x10^9/L)	6.1 (5.1, 8.0)	6.2 (4.9, 6.5)	6.6 (6.0, 7.1)	0.518
Neutrophils%	56.5 ± 7.4	61.3 ± 6.3	59.7 ± 4.1	0.291
Lymphocytes %	32.6 ± 6.5	30.2 ± 5.3	29.8 ± 4.7	0.54
Monocytes %	6.6 ± 1.5	6.1 ± 1.9	7.6 ± 3.2	0.444
Eosinophils %	3.4 (2.9, 3.6)	2.3 (1.4, 2.3)	2.2 (0.8, 3.2)	0.107
Basophils %	0.30 (0.20, 0.40)	0.39 (0.24, 0.6)	0.46 (0.39, 0.57)	0.296
Classical monocytes %	84.7 ± 5.7	83.2 ± 7.6	84.6 ± 6.2	0.857
Intermediate monocytes %	5.4 (3.7, 9.2)	3.8 (3.4, 6.1)	3.7 (3.2, 5.2)	0.509
Nonclassical monocytes %	7.0 ± 2.3	11.0 ± 5.8	9.8 ± 3.7	0.088

White blood cell subsets as a proportion of total white blood cells. Monocyte subsets as a proportion of total monocytes. Data are presented as mean ± SD for normally distributed data or median (Q1, Q3) for non-normally distributed data. ANOVA followed by post hoc Tukey's test was used for normally distributed datasets and Kruskall Wallis with Dunn's post hoc was used for non-normally distributed datasets. Abbreviations: WBC, white blood cells; SD, standard deviation.

### 3.3 Monocyte M1/M2 profile correlates with lipid profile

**Fig. 2:**
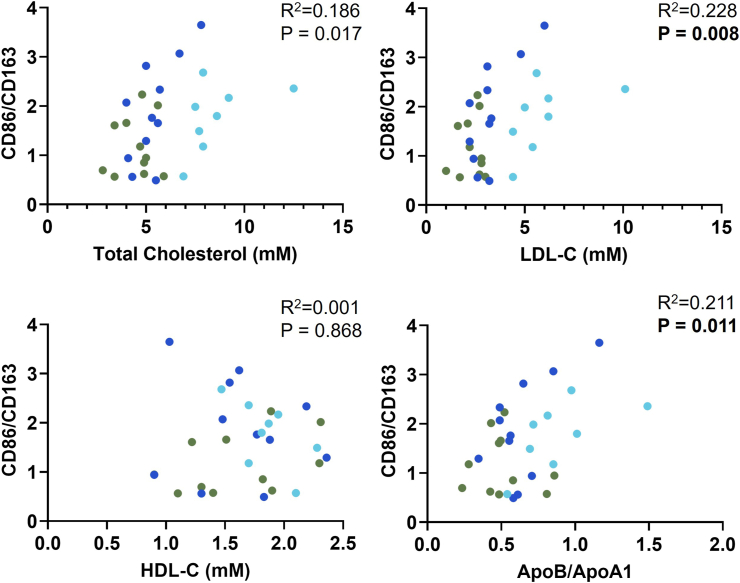
Correlation of monocyte M1/M2 ratio (CD86/CD163) with lipid factors. This data includes n = 30 (Controls n = 11, [olive], Untreated FH n = 8, [light blue], Treated FH n = 11 [dark blue]). Pearson correlation was used to determine whether correlations were significant and Pearson's R along with raw P-values are shown on the graphs. *p<0.0125* was accepted as significant after Bonferroni adjustment for multiple comparisons.

To assess whether lipid profile was related to the monocyte inflammatory profile, correlations were performed across the entire cohort (n = 30). We selected TC, LDL-C, HDL-C and ApoB/ApoA1 for correlation analysis and CD86/CD163 as a marker of overall monocyte inflammation. After Bonferroni correction, *p<0.0125* was considered significant. CD86/CD163 showed significant (moderate) positive correlations with LDL-C (*p = 0.008*), and ApoB/ApoA1 (*p = 0.011*) (Fig. 2) while the correlation with TC was not significant (*p = 0.017*) and no correlation was detected with HDL-C (*p = 0.868*) (Fig. 2).

### 3.4 FH monocytes readily formed M1 macrophages

**Fig. 3:**
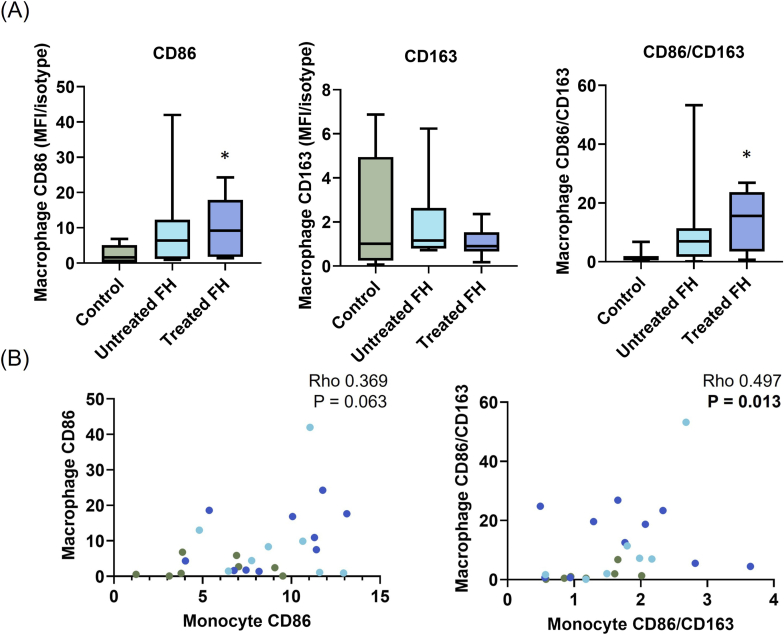
Monocyte-derived-macrophage expression of M1 and M2 markers (A) Monocytes were isolated and cultured with autologous serum for 7 days (Controls n = 8, Untreated FH n = 8, Treated FH n = 10). Marker expression on the macrophages was determined by flow cytometry. Markers are represented as median fluorescence intensity (MFI) of marker over MFI of isotype. Data are shown as box-and-whisker plots. Kruskall Wallis with post hoc Dunn's multiple comparison test was used for this non-normally distributed dataset. The statistical tests were run both with and without outliers and agreed on the differences between groups, so outliers were not removed. ∗*p<0.05* vs control ∗∗P < 0.01 vs control (B) Correlations were performed between marker expression of monocytes in whole blood and monocyte-derived macrophages cultured with autologous serum (Controls, n = 7 [olive], Untreated FH n = 7 [light blue], Treated FH n = 10 [dark blue]). Spearman rank correlation was used to determine whether correlations were significant. Spearman's Rho along with raw P-values are shown on the graphs. *p<0.025* was accepted as significant after Bonferroni adjustment for multiple comparisons.

Expression of the M1/M2 markers on macrophages showed variation between individuals. In macrophages cultured with autologous serum, CD86 was four times higher in the Treated-FH group than controls (Fig. 3A, *p = 0.026*) but, while CD86 appeared similarly elevated in Untreated-FH, it did not significantly differ from control levels (*p = 0.166*). The M2 marker CD163 showed no differences between groups (*p = 0.523*). The M1/M2 ratio (CD86/CD163) was over seven-fold higher in the treated group than controls (*p = 0.0168*). Again, while this appeared elevated in Untreated-FH compared to controls, the difference was not significant (*p = 0.151*). High levels of CD86/CD163 on monocytes associated with high CD86/CD163 on macrophages (*p = 0.013*, Fig. 3B). CD86 of monocytes also showed a trend for a positive association with macrophage CD86 (*p = 0.063*). This indicates that, at a cohort level, the degree of monocyte inflammation moderately correlates with that of macrophage inflammation.

## 4 Discussion

This study indicates that FH, even when statin-treated, is associated with inflammatory M1-skewing of monocytes. M1-skewing correlated with high LDL-C and ApoB/ApoA1 levels which may indicate lipid factors influence inflammation. Furthermore, monocytes from FH readily become M1 macrophages. We propose that the presence of FH results in M1-skewed monocytes with greater propensity to form inflammatory M1 macrophages, even with moderate LDL-C reduction. This could promote M1 macrophage formation in the atherosclerotic plaque and contribute to plaque instability and heightened CVD event risk.

That the groups were well-matched indicates that age and sex are not likely to contribute to differences between groups. The overrepresentation of females within our cohort was unexpected, as FH has similar prevalence in males and females [21]. That men generally consult less for primary healthcare [22] may have contributed to this disparity. Regardless, equal representation of males and females would be preferable. Statin treatment appeared effective, with LDL-C levels 44 % lower in the treated compared to untreated group. However, it is unlikely that lipid targets for these individuals had been met. Initial targets are a 50 % reduction in LDL-C, with an LDL-C goal <1.8 mmol/L [6]. Therefore, the findings for this cohort may not represent a cohort that was meeting cholesterol targets.

The similar leukocyte profile between FH and controls agrees with many studies [17,[23], [24], [25]]. Though others report small elevations in monocytes [25] or neutrophils [23] in FH. Similarly, while we and others found similar monocyte subset proportions in FH and controls [16,17,23], some have reported elevations in intermediate or nonclassical monocytes [25,26]. The high CD86 in both FH groups indicates inflammatory M1-skewed monocytes. This aligns with a study reporting elevated “M1 monocytes” in FH, though their approach differed [16]. Combining these findings indicates a greater number of inflammatory monocytes and a greater degree of inflammation across all monocytes. The clinical implications of enhanced M1 monocyte skewing are not yet established, but elevated inflammatory functions are likely. We have shown M1 monocyte skewing in CVD and a correlation between CD86/CD163 ratio and inflammatory cytokine release, evidence of these markers being linked to inflammatory functions [14]. M1-skewing, shown by high CD86 expression, could therefore indicate enhanced inflammatory functions in the circulation. The trend for higher CD11b in the FH groups appears contradictory to its classification as an M2 marker [27]. However, others report high CD11b in FH [17,23,28] to indicate heightened monocyte activation or adhesion. That monocyte profile was inflammatory in both Treated and Untreated FH individuals hints that lipid-lowering does not resolve these inflammatory changes. This mirrors the findings of others examining different aspects of monocyte inflammation. For example, statin treatment of three months’ duration in FH did not reverse monocyte activation (high CD11b, CD11c) or the elevated inflammatory cytokine release [17], and in people with CVD, PCSK9 antibody achieved LDL-C lowering but did not improve monocyte inflammatory gene expression [24]. On the other hand, statin treatment did reduce selected adhesion and migration markers (CCR2 and CD29) [17]. Overall, while statins certainly lower LDL-C, they appear to have limited impact on monocyte inflammatory profile.

That LDL-C and ApoB/ApoA1 significantly correlated with M1/M2 ratio indicates that high LDL-C (and ApoB) could be driving monocyte inflammation. Considering others found that both pre-treatment and on-treatment LDL-C correlated with the proportions of “M1” relative to “M2 monocytes” [16], high LDL-C may promote a greater number of “M1 like” cells, as well as a greater degree of inflammation on all monocytes. This is in addition to other monocyte markers, such as CCR2-a migration marker, that have been shown to correlate with LDL-C [23]. If the high LDL-C of FH is causing inflammation, then why does inflammation not subside with LDL-C lowering? There are at least two likely explanations. One is that the changes induced by high LDL-C are permanent. Changes that persist despite lipid lowering are proposed to be evidence of trained immunity, reprogramming of monocyte precursors in the bone marrow [17]. This explains how changes could persist even though monocytes have a lifespan of less than 1 week [29]. However, changes could be long-lived rather than permanent. If so, extended or more aggressive treatment could lead to improvements in monocyte inflammation and subsequently that of their derived macrophages. There is evidence that some treatments attenuate monocyte inflammation. For example, in LPS-stimulated monocytes, TNFα production was improved after 24 weeks of PCSK9 mAB treatment [23] whereas LDL-apheresis reduced CD11b and CD11c expression [30]. Regardless of whether monocyte inflammation is permanent or long-lived, it has potential to exacerbate development of atherosclerosis for the duration it persists. Thus, a deeper understanding of how monocyte inflammation drives CVD, and whether direct targeting of inflammation attenuates this, is crucial. This must also be considered in the context that few people with FH are diagnosed and adequately treated [31] and likely have the combined impact of high LDL-C and inflammation contributing to atherosclerosis progression.

The higher CD86 and CD86/CD163 on the macrophages of the Treated-FH group suggest enhanced formation of inflammatory M1 macrophages from FH monocytes. That this was not significant in the untreated group may, at first glance, indicate that statins promote formation of M1 macrophages. However, the pattern of expression across the groups indicates the more likely explanation for a significant difference being limited to the treated group is the study being powered to detect large differences. It could therefore not consistently detect effects below this level. The correlation between CD86/CD163 levels on monocytes and macrophages should be interpreted with caution. While the relationship exists at a cohort level, the wide spread of data indicates that a direct relationship between monocyte and macrophage profile is not present in everyone. Nevertheless, the correlation hints that monocyte skewing influences macrophage profile. Others have shown a similar phenomenon, where human monocytes with lower M2 markers were less able to form M2 macrophages in culture [15]. As the environment also influences macrophage formation [32], components within autologous serum may also contribute to enhanced M1 macrophage formation in FH. In vitro, LDL-C increased the inflammatory cytokine release of monocyte-derived M1 macrophages while reducing anti-inflammatory functions of M2 macrophages [33]. Considering that our Treated-FH group had similar LDL-C levels to controls, then monocyte skewing is likely a greater contributor to macrophage phenotype than the presence of serum. Importantly, the combination of ex vivo monocytes and autologous serum is a reasonable representation of the FH environment in vivo. It is therefore likely that FH would be accompanied by increased presence of M1 macrophages in atherosclerotic plaques. As M1 retention in atherosclerotic plaques is associated with plaque progression [34] and CD86 is directly associated with plaque instability [12], monocyte/macrophage profile could be an important contributor to risk of AMI in FH individuals.

That serum cytokines did not differ between groups indicates that systemic inflammation is not increased in this FH cohort. This is a somewhat contentious topic. In statin-treated FH adults (n = 14) with good LDL-C reduction, serum levels of inflammatory (TNFα, IL-1β IL-6, hsCRP) and anti-inflammatory (IL-10) cytokines showed no difference from controls [35]. In stark contrast, homozygous FH individuals (n = 10, 9–28 years of age) had elevated inflammatory (TNFα, IL-1β, IL-6, and hsCRP) and anti-inflammatory (IL-10) cytokines compared to controls [36]. Many studies fall somewhere between these extremes, with older age [37] and statin treatment [38] likely masking cytokine differences in FH. It is important to note that while monocytes produce cytokines, their cytokine production cannot be inferred from serum cytokine levels. While we did not examine whether monocytes in FH produced more inflammatory cytokines, other studies show this may [39] or may not be [40] the case. Further studies examining monocyte or PBMC production of cytokines would be informative.”

As with any study, this work has limitations. The study being powered to detect large differences means that any lack of observed difference does not confirm that no difference exists. Differences that exist but are below the detection level would be missed. If a smaller effect size than we observed was found to be clinically relevant, the findings would need to be revisited with a larger sample size. This would also permit us to examine probable compared to definite FH as well as other subgroup analysis. While we suggest that treatment does not improve monocyte inflammation, the use of two different groups is imperfect. Even with similar demographics, the findings would be stronger if examined in the same individuals pre- and post-treatment. Here, FH diagnosis was determined clinically rather than by genetic testing. Utilising a clinical diagnosis risks the “FH” groups including people with polygenic hypercholesterolaemia, which is difficult to distinguish from FH [41,42]. But people with clinical features do not always carry a recognised mutation [43]. Conversely some people with an FH-causative mutation only have mild clinical presentations [44,45]. Neither approach is perfect and, ideally, they would be used in combination [46].

This study reveals pathways for further investigations. The monocyte inflammatory changes were limited to specific markers and the pathways responsible are therefore unclear, opening this to investigation. Studies examining different types, doses and durations of statins may clarify whether any specific statin regime could achieve reduction in monocyte inflammation. Studies could also expand this to other treatment types. To gain clarity on whether macrophage inflammation was predominantly due to intrinsic factors (skewing) or environment, control and FH monocytes could be cultured with human (blood type) AB serum (i.e. serum that lacks the antibodies for A and B blood). If LDL-C lowering cannot resolve inflammation, the next question concerns targeted inflammatory treatments. Colchicine and canakinumab have been assessed in clinical trials and were successful in reducing CVD events [47,48]. They may be able to improve monocyte inflammatory profile. In mice, colchicine mitigated monocyte recruitment and reduced inflammatory monocyte content of atherosclerotic plaques [49]. In vitro, human monocytes treated with colchicine had reduced chemokine gene expression [50]. Colchicine treatment in humans reduced circulating monocytes and altered other immune cells [51]. It has been proposed that future research should aim to develop therapies directly targeting inflammation [51]. To this end, linking inflammatory alterations to CVD outcomes, and determining which alterations can be improved with targeted treatment, would be valuable for both FH and the broader population at risk of CVD.

In conclusion, FH individuals have M1-skewed monocytes which readily became M1 macrophages in culture. This indicates that FH monocytes are circulating in a more inflammatory state, which could contribute to an increased presence of M1 macrophages in atherosclerotic plaques. This could lead to atherosclerotic plaque instability as plaques form and progress. Inflammatory alterations in monocytes of FH individuals could therefore contribute to their increased CVD risk. Importantly, this indicates monocyte M1/M2 profile could be a biomarker that shows mechanistic implications for inflammation in CVD.

## Author contributions

Helen Williams: Conceptualisation, Formal analysis, Funding acquisition, Investigation, Methodology, Project administration, Resources, Supervision, Visualisation, Writing - original draft, Writing-review & editing Habib Francis: Formal analysis, Investigation, Writing - original draft, Writing - review & editing Jasmin Huang: Formal analysis, Investigation, Writing – original draft, Writing - review & editing Rana Baraz: Investigation, Resources, Writing - review & editing Rekha Marimuthu: Investigation, Methodology, Writing - review & editing Heather Medbury: Conceptualisation, Funding acquisition, Methodology, Project administration, Resources, Supervision, Writing - review & editing Stephen Li: Conceptualisation, Funding acquisition, Investigation, Project administration, Resources, Supervision, Writing - review & editing.

## Data availability statement

Datasets are available on request: The raw data supporting the conclusions of this article will be made available by the authors, without undue reservation.

## Funding sources

Funding was provided by NSWHP-ICMPR-Westmead Private Practice Fund and Westmead Hospital-WSLHD, NSW Health.

## Declaration of competing interest

The authors declare that they have no known competing financial interests or personal relationships that could have appeared to influence the work reported in this paper.
