# Supplementary material for: Monocytes from people with Familial Hypercholesterolaemia are inflammatory, despite statin-treatment
Source: Atheroscler Plus. 2025 Sep 10;61:73–81. doi: 10.1016/j.athplu.2025.09.002 (PMC12496229; doi:10.1016/j.athplu.2025.09.002)
Supplement: Multimedia component 1 [file mmc1.docx]

Supplementary Material

**Supplementary Table 1:** List of lipid-lowering medication taken by Treated FH group

| Participant number | Statin name / dosage | Duration of current treatment |
| --- | --- | --- |
| 1 | Atorvastatin 40 | > 2 months |
| 2 | Atorvastatin 40 | >2 months |
| 3 | Atorvastatin 80/Ezetimibe 10 | >2 months |
| 4 | Rosuvastatin 10/ Ezetimibe 10 | >2 months |
| 5 | Rosuvastatin 20 | >2 months |
| 6 | Rosuvastatin 20 | 5 years |
| 7 | Rosuvastatin 20 | 5 months |
| 8 | Rosuvastatin 40 | 5 years |
| 9 | Simvastatin 20/Ezetimibe 10 | >2 months |
| 10 | Simvastatin 40 | >2 months |
| 11 | Simvastatin 80/Ezetimibe 10 | 12 months. |

Note: many participants had used other statins prior to current treatment regime.

**Supplementary Table 2:** Serum cytokine levels determined by Luminex assay

| Conc (pg/mL) | Control (n=6) | Untreated FH (n=6) | Treated FH (n=9) | Global *p=* |
| --- | --- | --- | --- | --- |
| IFNγ | 95.0 (42.1,  125.1) | 24.1 (8.2, 40.4) | 37.1 (14.2, 98.1) | 0.241 |
| IL-1β | 15.1 ± 21.8 | 0.5 ± 1.0 | 0.6 ± 2.0 | 0.952 |
| IL-6 | 144.6 ± 104.2 | 88 ± 60.3 | 78.1 ± 50.6 | 0.303 |
| IL-8 | 38.5 (30.0, 67.3) | 24.7 (14.0, 38.7) | 21.2 (12.2, 38.3) | 0.288 |
| IL-12p70 | 88.8 ± 115.9 | 161.7 ± 161.4 | 36.5 ± 61.2 | 0.373 |
| IL-17A | 55.6 (46.4, 70.8) | 26.2 (22.8, 31.3) | 42.6 (8.2, 82.5) | 0.559 |
| MCP-1 | 556.8 ± 196.2 | 486.5 ± 383 | 623.7 ± 289.6 | 0.686 |
| TNFα | 27.2 ± 47.6 | 11.1 ± 3.7 | 12.0 ± 7.8 | 0.337 |
| IL-10 | 6.8 (0.7,13.9) | 39.2 (21.6, 56.9) | 10.9 (10.2, 12.4) | 0.736 |
| IL-1RA | 185.4 ± 191.9 | 212.4 ± 245.8 | 61.4 ± 60.5 | 0.254 |
| IL-4 | 25.9 ± 43.8 (6,1-49.9) | 21.6 (8.6, 80.6) | 12.9 (7.8,22.7) | 0.879 |

M1 (white) and M2 (grey) related cytokines measured in serum by Luminex assay. Data are presented as mean ± SD for normally distributed data or median (Q1, Q3) for non-normally distributed data. ANOVA followed by post hoc Tukey's test was used for normally distributed datasets and Kruskall Wallis with Dunn’s post hoc was used for non-normally distributed datasets. Abbreviations: IFN, interferon; IL, interleukin; MCP, monocyte chemoattractant protein; TNF, tumor necrosis factor.

**
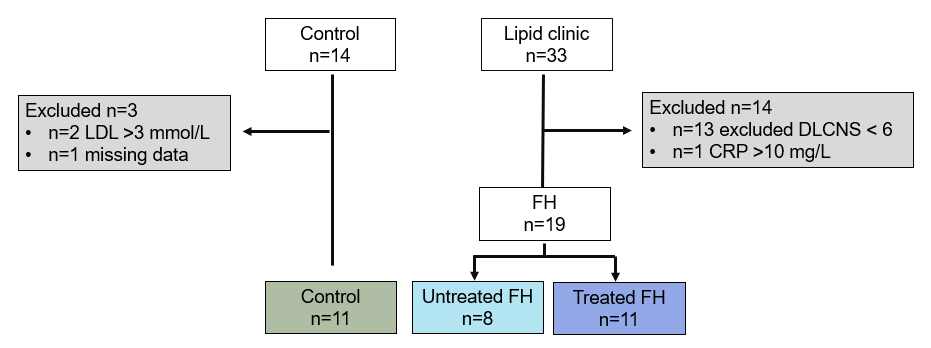
Supplementary Figure 1**: CONSORT flow diagram of participant recruitment, exclusion, and final cohort. Abbreviations: CRP, C-reactive Protein; DLCNS, Dutch lipid clinic network score; FH, Familial hypercholesterolaemia; LDL, Low-density lipoprotein.

**
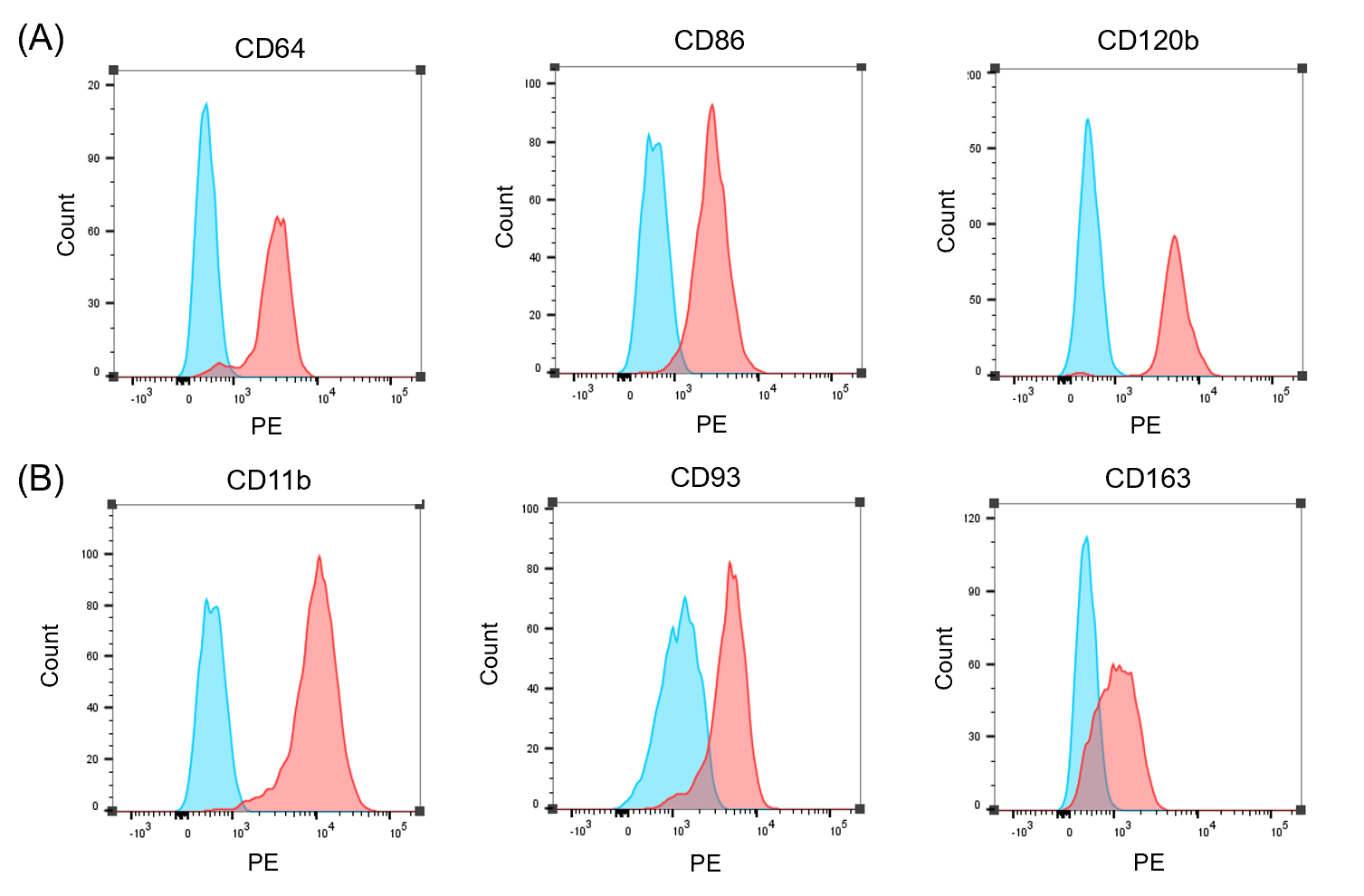
**

**Supplementary Figure 2:** Monocyte expression of **A** M1 and **B** M2 markers. Representative shifts of each marker (red) against isotype control (blue).

**
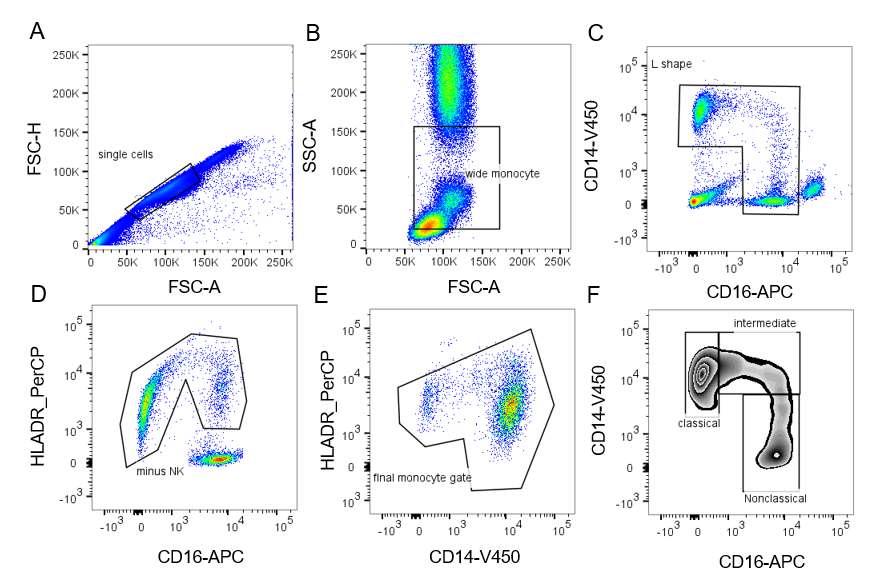
**

**Supplementary Figure 3:** Monocyte gating **(A)** “single cells” gate to remove doublets and debris **(B)** “wide monocyte” gate to exclude the majority of granulocytes and lymphocytes **(C)** inverted “L shape” gate to select monocytes on CD14/CD16 expression and exclude other cells **(D)** “minus NK” gate to exclude natural killer cells based on low HLADR **(E)** “final monocyte gate” excluding B cells based on high HLADR **(F)** The three monocyte subsets “classical” “intermediate” and “nonclassical”.
